# Temperature-Dependent Phonon Scattering and Photoluminescence in Vertical MoS_2_/WSe_2_ Heterostructures

**DOI:** 10.3390/nano13162349

**Published:** 2023-08-16

**Authors:** Wajid Ali, Ye Liu, Ming Huang, Yunfei Xie, Ziwei Li

**Affiliations:** 1 Hunan Institute of Optoelectronic Integration, College of Materials Science and Engineering, Hunan University, Changsha 410082, China; wajidali@hnu.edu.cn (W.A.);; 2 Wuhan National Laboratory for Optoelectronics, School of Physics, Huazhong University of Science and Technology, Wuhan 430074, China

**Keywords:** transition metal dichalcogenides, heterostructures, phonon scattering, thermal quenching, thermal activation energy

## Abstract

Transition metal dichalcogenide (TMD) monolayers and their heterostructures have attracted considerable attention due to their distinct properties. In this work, we performed a systematic investigation of MoS_2_/WSe_2_ heterostructures, focusing on their temperature-dependent Raman and photoluminescence (PL) characteristics in the range of 79 to 473 K. Our Raman analysis revealed that both the longitudinal and transverse modes of the heterostructure exhibit linear shifts towards low frequencies with increasing temperatures. The peak position and intensity of PL spectra also showed pronounced temperature dependency. The activation energy of thermal-quenching-induced PL emissions was estimated as 61.5 meV and 82.6 meV for WSe_2_ and MoS_2_, respectively. Additionally, we observed that the spectral full width at half maximum (FWHM) of Raman and PL peaks increases as the temperature increases, and these broadenings can be attributed to the phonon interaction and the expansion of the heterostructure’s thermal coefficients. This work provides valuable insights into the interlayer coupling of van der Waals heterostructures, which is essential for understanding their potential applications in extreme temperatures.

## 1. Introduction

Transition metal dichalcogenides have recently garnered significant interest due to their scalability and thickness-dependent electrical and optical properties [1]. The evolution of bulk TMDs to atomically thin 2D layered structures is characterized by their transition from indirect to direct bandgap semiconductors [2]. Such a transition of bandgaps has significant implications for their optoelectronic properties and potential applications [3,4,5,6]. These 2D semiconducting materials, MX_2_ (M = Mo, W; X = S, Se), are exemplified by compounds such as molybdenum disulfide (MoS_2_) and tungsten diselenide (WSe_2_) with direct bandgaps of 1.9 eV and 1.6 eV, respectively [7]. Beyond monolayers, vertically-stacked heterostructures based on the van der Waals force provide a fascinating platform for investigating novel physical phenomena [8]. With the assistance of an artificial stacking arrangement, electronic band engineering of heterostructures can be achieved, resulting in the modulation of optical properties via interlayer coupling effects [9].

Heterostructures composed of two-dimensional materials have been shown to exhibit various intriguing phenomena, including ultrafast charge transfer [10], high PL quantum yield [11], and interlayer valley physics [12]. These properties make them potential candidates for applications in field-effect transistors [13], electrocatalysis [14], and photodetectors [15]. The thermodynamic stability of these heterostructures plays a critical role in their optoelectronic properties [16]. A comprehensive understanding of the correlation between phonon scattering and heterostructure interfacial coupling is imperative to achieve the optimal performance of such structures [17,18]. Raman spectroscopy is an essential tool to investigate the interaction between electrons and phonons in 2D materials and their heterostructures [19]. Research on temperature-dependent Raman spectroscopy of few-layered WS_2_ provides valuable insights into their intrinsic phonon scattering processes and thermal properties [20]. Several early experiments investigated the impact of temperature on the phononic properties of few-layered TMDs, including MoS_2_, WS_2_, MoSe_2_, and others [21]. These studies have demonstrated that temperature-dependent behaviors of phononic modes in these nanosheets are attributed to the effects of thermal expansion and anharmonic resonance [22]. Moreover, some studies investigated the impact of interlayer coupling on the Raman spectra of TMDs heterostructures, which demonstrated a strong dependence on the stacking orientation of the constituent monolayers [23,24].

This work provides a systematic investigation into the temperature-dependent Raman characteristics of a heterostructure composed of MoS_2_ and WSe_2_ monolayers. Raman spectroscopy measurements were conducted over the controlled temperature range of 79 to 473 K to investigate the in-plane ($E_{2g}^{1}$) and out-of-plane ($A_{1g}$) vibrational modes of the heterostructure. Experimental observations indicate that both Raman modes display a nearly linear dependence across the entire temperature range, alongside a commensurate variation in their full width at half maximum. The primary focus of this study is on attempting to understand the variation of interlayer interactions, particularly temperature-dependent phonon interactions, and the thermal expansion coefficients of the heterostructure under high vacuum conditions. Furthermore, temperature-dependent photoluminescence was also observed and utilized to determine the activation energy of the PL emissions caused by thermal quenching. This work provides a platform to investigate the photodynamics of low-dimensional materials and also helps to understand the electronic and photonic properties of van der Waals heterostructures.

## 2. Materials and Methods

The synthesis of 2D transition metal dichalcogenides has revolutionized modern techniques, such as physical vapor deposition (PVD) [25], chemical vapor deposition (CVD) [26], electrodeposition [27], and thermal synthesis [28], facilitating their advancement and enhancing their capabilities for diverse applications. In this work, a MoS_2_ monolayer was grown by a chemical vapor deposition method using sulfur (S) and molybdenum oxide (MoO_3_) powders. MoO_3_ and S powder were put in separate boats at the center of a fused quartz tube located in a furnace. Subsequently, a SiO_2_/Si wafer was suspended on one of the boats, and the temperature was ramped up to 810 °C for 30 min and maintained for 10 min. When it cooled down to room temperature, triangular-shaped MoS_2_ monolayers were successfully prepared. Further details can be found in our previous work [29]. The synthesis procedure was conducted at atmospheric pressure using Ar gas as a carrier agent with a flow rate of 70 sccm. The same CVD procedure was employed to grow WSe_2_ utilizing tungsten oxide (WO_3_) and selenium (Se) powder as source materials, with the furnace temperature kept constant at 950 °C and naturally allowed to cool. A comprehensive description of the synthesis procedures and a schematic depiction of the CVD setup can be found in Supplementary Section S1. A simple wet transfer technique was employed to prepare the MoS_2_/WSe_2_ heterostructures [30]. Further details about the transfer process and the schematic illustration can be found in Supplementary Section S2. The heterostructure underwent annealing in a vacuum furnace at 70 °C for 12 h to eliminate water molecules and promote better crystallinity.

For spectral measurements of materials, PL and Raman spectra were analyzed using an iHR550 Raman spectrometer from Horiba with a laser excitation of 532 nm. An objective lens providing 50 times magnification was utilized to enable a more detailed analysis of the sample’s features. The diameter of the laser spot was about 1 µm. The spectrometer was equipped with gratings of 300 g mm^−1^ and 1200 g mm^−1^ to facilitate accurate analysis of the spectral data. Furthermore, a temperature-controlled cryostat (THMS-600, Linkam Scientific Instruments Ltd., Redhill, UK) was utilized to investigate the temperature-dependent behavior of the heterostructure (Supplementary Section S3). The cryostat utilized liquid nitrogen to attain the lowest temperature of the boiling point of liquid nitrogen. Samples were loaded into the cryostat full of nitrogen to avoid any impact from the surface adsorption of gas molecules and to enable precise temperature control during measurements.

## 3. Results and Discussion

**Figure 1:**
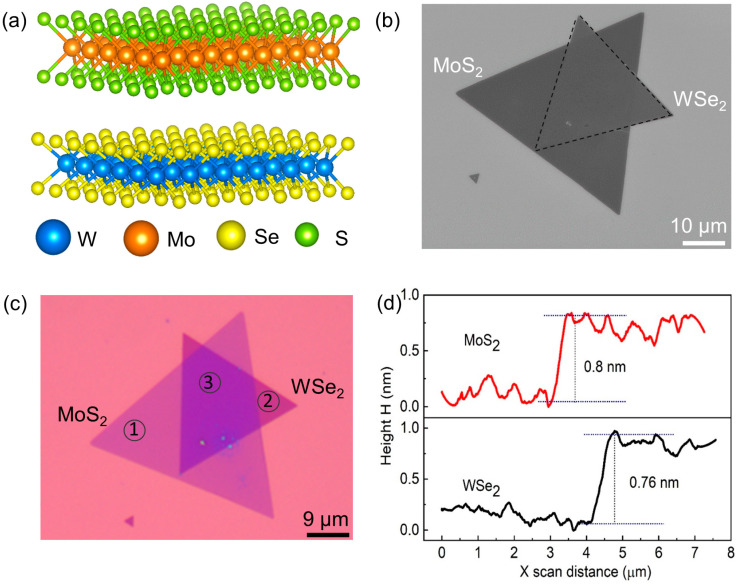
Structure and morphology of MoS_2_/WSe_2_ heterostructure. (**a**) Schematic of MoS_2_ and WSe_2_ monolayers. (**b**) SEM and (**c**) optical images of the vertically stacked heterostructure with scale bars of 10 and 9 µm, respectively. (**d**) Height profiles of MoS_2_ and WSe_2_ monolayers confirmed by AFM.

Figure 1a shows the schematic view of the MoS_2_/WSe_2_ heterostructure. The MoS_2_ and WSe_2_ monolayers were in a 2H phase, behaving as semiconductors with direct band gaps. Figure 1b shows the scanning electron microscopy (SEM) image of the as-prepared MoS_2_/WSe_2_ heterostructure, where the top WSe_2_ monolayer has been transferred onto the bottom MoS_2_ monolayer. The scale bar is 10 µm. Figure 1c shows the optical image of heterostructures, where monolayer and heterostructure regions can be observed. The monolayer thicknesses of MoS_2_ (region 1) and WSe_2_ (region 2) were determined to be 0.8 nm and 0.76 nm, respectively, using atomic force microscopy (AFM) as described in Supplementary Section S1. The height profiles of both monolayer regions are plotted in Figure 1d. Additional AFM results of the heterostructure (region 3) with a 0.39 nm interlayer distance can be found in Supplementary Section S1.

**Figure 2:**
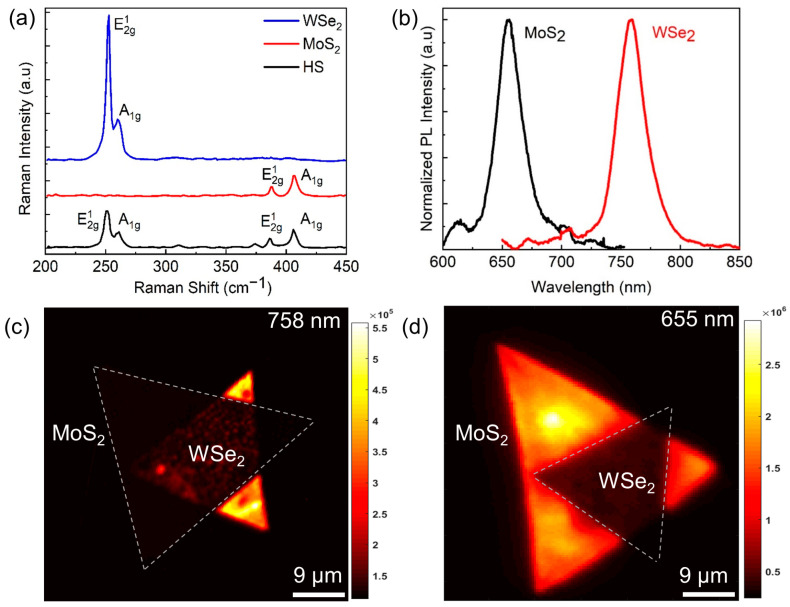
Raman and PL spectra of MoS_2_/WSe_2_ heterostructures. (**a**) Raman shifts of MoS_2_ and WSe_2_ monolayers and their heterostructures. (**b**) PL spectra of MoS_2_ and WSe_2_ monolayers with the main peaks at 655 nm and 758 nm, respectively. Mapping images of PL intensity of heterostructure samples at a wavelength of (**c**) 758 nm and (**d**) 655 nm.

Raman spectroscopy was employed to characterize the structure and spectral properties of the MoS_2_/WSe_2_ heterostructure. The sample was put into a vacuum annealing furnace to anneal the heterostructure sample and mitigate the influence of defects and gas adsorption (Supplementary Section S2). Moreover, to avoid any local heating of the sample, which can alter peak positions in the Raman spectra, the excitation power was carefully controlled at 1.2 mW. The Raman spectra in Figure 2a display two distinct peaks corresponding to the longitudinal (*E*) and transverse (*A*) modes of monolayer regions in the MoS_2_/WSe_2_ heterostructure. The Raman modes of $E_{2g}^{1}$ and $A_{1g}$ for MoS_2_ (red curve) show a separation of about 18 cm^−1^, which is consistent with previously reported works on monolayer MoS_2_ [31].

The transverse and longitudinal modes were detected at 252 cm^−1^ and 260 cm^−1^, respectively, in the monolayer WSe_2_ region of the heterostructure, consistent with previous studies [32]. The Raman spectra acquired from the heterostructure region (black curve) shown in Figure 2a exhibit identical vibrational modes, as observed in both monolayers. Nevertheless, a marginal reduction is observed in the intensity of these modes, which is attributable to enhanced excitation light scattering caused by the presence of the heterostructure. The MoS_2_/WSe_2_ heterostructure manifests a type II band alignment, which facilitates effective charge transfer, yet concurrently reduces the PL intensity by spatially separating the valence and conduction bands. The photoluminescence peaks exhibited by the heterostructure in Figure 2b are attributed to the “A” excitons, corresponding to the direct bandgap transitions at the K-point of the Brillouin zone in both MoS_2_ and WSe_2_. For the MoS_2_ spectrum, an additional peak at higher energy near 2.02 eV was observed, corresponding to the “B” exciton formed due to transitions between the spin-orbit split valence band and the conduction band [33]. However, the emission strength of the B exciton is relatively weaker than the A exciton due to its lower oscillator strength, which resulted from an indirect transition.

PL mapping was performed at specific wavelengths to assess the photoemissions of MoS_2_/WSe_2_ heterostructures. As illustrated in Figure 2c,d, the resulting PL emission maps at 758 nm and 655 nm provide valuable insight into the distribution of PL intensity from MoS_2_ and WSe_2,_ respectively, in the heterostructure. Remarkably, the photoluminescence maps obtained in this study demonstrate a stronger emission signal emanating from the MoS_2_ monolayer, which can be ascribed to its elevated radiative recombination rate and binding energy. Importantly, these findings align with previous investigations of similar systems [34]. Moreover, photoluminescence measurements were conducted for both MoS_2_ and WSe_2_ in the monolayer position as well as the heterostructure region. Notably, the peak intensities at 655 nm and 758 nm in the heterostructure’s region 3 exhibit significant quenching compared to the monolayer regions (1 and 2). This behavior can be attributed to the ultrafast charge transfer phenomenon observed in atomically thin MoS_2_/WSe_2_ heterostructures. Detailed discussions can be found in Supplementary Section S4 for a more in-depth analysis of these findings. All Raman and PL spectra in Figure 2 were measured at room temperature using a 532 nm laser of 1.2 mW.

**Figure 3:**
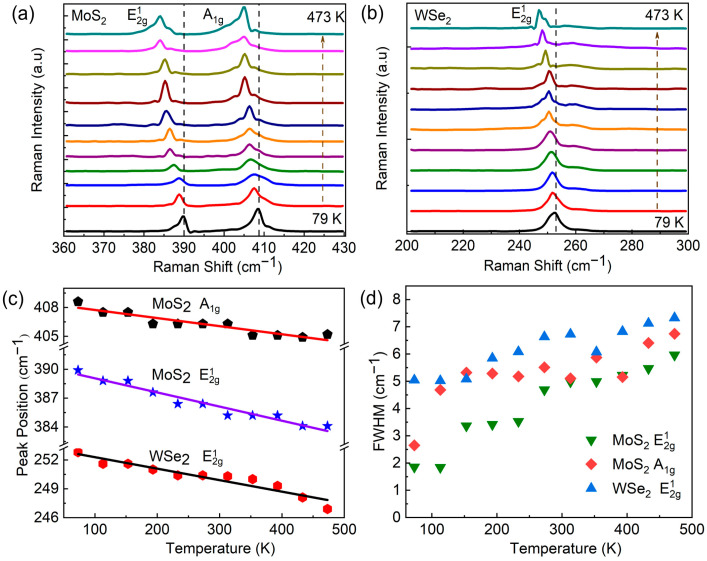
Temperature-dependent Raman spectra of MoS_2_/WSe_2_ heterostructures. (**a**,**b**) The variation of Raman modes of MoS_2_ and WSe_2_ at varying temperatures of 79–473 K. The vertically black and orange dashed lines are drawn to highlight specific Raman modes and indicate temperature increase, respectively. (**c**) Temperature-induced Raman shifts of three characteristic peaks showing linear fitting curves. (**d**) The full width at half maximum of Raman peaks at different temperatures.

To better comprehend the vibrational modes and interlayer coupling in MoS_2_/WSe_2_ heterostructures, temperature-dependent Raman measurements ranging from 79 to 473 K, as illustrated in Figure 3a,b, enable insights into their thermal behavior. The Raman findings indicate strong scattering intensities of the $E_{2g}^{1}$ and $A_{1g}$ modes for MoS_2_/WSe_2_ heterostructures with increasing temperatures. Thermal expansion of the lattice is known to trigger a decline in the vibrational frequency with increasing temperature, thereby inducing a downward shift of the Raman modes towards lower frequencies, as corroborated by the outcomes shown in Figure 3a,b. Remarkably, the uniform redshift across all Raman modes with increasing temperature implies a systematic alteration in the vibrational characteristics of the heterostructure. At high temperatures, it looks as though split peaks appear apart from the main peaks, but actually, we are observing the temperature-dependent enhancement and widening of inconspicuous peaks at low temperatures. To visualize these changes, Figure 3c illustrates the temperature-dependent peak positions of the Raman modes. Our experimental data exhibit a linear decline with temperature and are well-fitted by a linear function.

Elevated temperatures lead to increased atomic vibrations in the lattices of WSe_2_ and MoS_2_, which causes an increase in the scattering rate and a reduction in the lifetime of vibrational modes. These effects contribute to the broadening of the Raman spectra, as demonstrated by the increase in full width at half maximum (FWHM) observed in Figure 3d. The FWHM of the WSe_2_ region in the heterostructure is particularly significant, perhaps due to its lower binding energy, which leads to the higher scattering of lattice modes at higher temperatures (indicated in Figure 3d). When the temperature rises, the thermal energy of the lattice vibrations increases, resulting in an increased number of phonon scattering events. In addition, the thermal energy of the lattice vibrations in 2D materials also increases, resulting in a greater number of phonon scattering events. This, in turn, leads to an increase in the FWHM of the Raman peak associated with the $A_{1g}\;$mode. In MoS_2_, this effect is more pronounced for the $A_{1g}$ mode compared to the $E_{2g}^{1}$ mode due to the latter’s less sensitive in-plane vibrations of Mo and S atoms, as illustrated in Figure 3d. It is noteworthy that slight deviations observed at certain temperatures may be attributed to slight instrumentation instability in our Raman setup. The differences in Raman peak broadening between the $E_{2g}^{1}$ and $A_{1g}$ modes of WSe_2_ and MoS_2_ provide valuable information about the behavior of these materials under varying thermal conditions.

**Figure 4:**
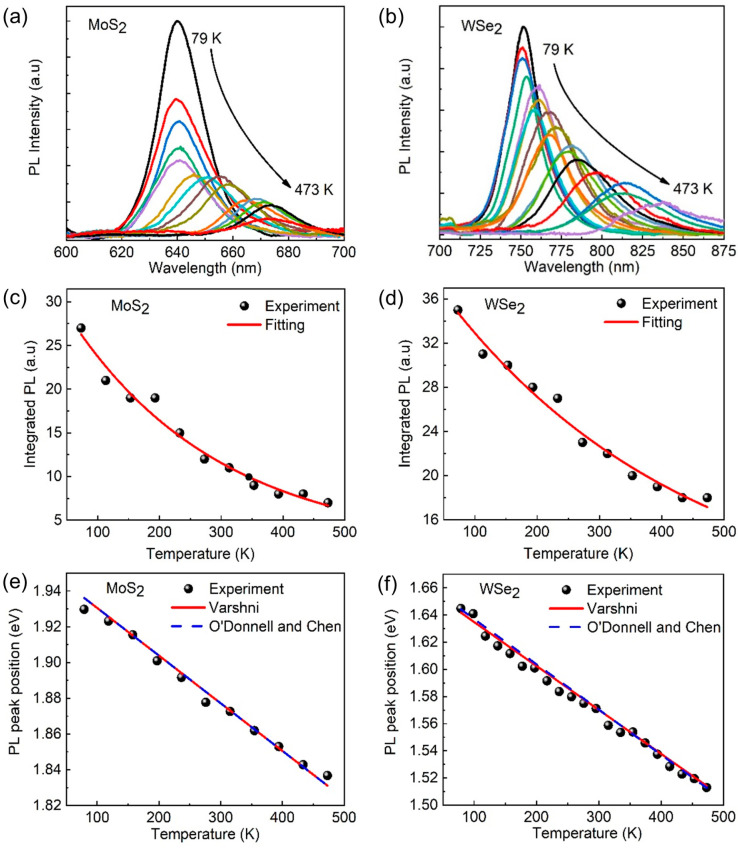
Temperature-dependent photoluminescence. (**a**,**b**) PL spectra of MoS_2_ and WSe_2_ at various temperatures. The peak position shifts red, and the intensity decreases as temperature increases form 79 to 473 K. (**c**,**d**) Integrated PL intensities of corresponding regions of MoS_2_ and WSe_2_, respectively. Solid lines depict experimental data, while the scattered points show the corresponding Arrhenius fitting. (**e**,**f**) Temperature-dependent shifting of PL peaks detected for MoS_2_ and WSe_2_ regions in the heterostructures. Red lines and dotted blue lines represent fitting curves for Equations (2) and (3).

In addition to its effects on the Raman spectra, temperature can also influence the photon emissions of the MoS_2_/WSe_2_ heterostructures. The aspect of temperature-dependent photoluminescence in heterostructures is crucial in device physics to improve their operation performance. The combined effect of thermal and optical energies that contribute to the de-trapping of carriers and desorption of adsorbates (such as O_2_ and H_2_O) could result in changes in the conductivity of the material, as well as the optical emission properties, as discussed elsewhere [35]. In the case of heterostructures, temperature-dependent PL spectroscopy has revealed new and interesting effects, including an anomalous redshift, which could be related to thermal quenching, defect engineering, and strain. Several studies have explored and linked the temperature-dependent PL spectra to the underlying band structure, electronic properties, and potential device applications of heterostructures. To investigate thermal quenching and the underlying band structure, photoluminescence measurements of the heterostructure were conducted over a range of temperatures (79 K to 473 K), as illustrated in Figure 4. The results of the photoluminescence measurements reveal a significant temperature dependence in both the peak position and intensity of the spectrum. As the temperature increases, both the WSe_2_ and MoS_2_ peaks experience redshifts towards longer wavelengths, as demonstrated in Figure 4a,b. The reduction in photoluminescence intensity with increasing temperature is a well-known phenomenon observed in both two-dimensional transition metal dichalcogenides and bulk semiconductors, attributable to the amplification of non-radiative recombination, thermal activation of electrons and holes, and phonon-assisted recombination.

Here, the investigation of the temperature dependence of photoluminescence intensity in MoS_2_/WSe_2_ heterostructures revealed that the Arrhenius formula (Equation (1)) provides a good fit for the experimental data. This formula correlates the photoluminescence quenching rate with the thermal activation energy for non-radiative recombination, as demonstrated in Figure 4c,d. These findings offer insights into the fundamental processes involved in optoelectronic devices and emphasize the significance of carefully controlling temperature effects in such systems [36].

(1) $$I\left(T\right)=\frac{A}{1+C\;e^{\left(-E/k_{B\;}T\right)}}\;$$

Here, in Equation (1), the temperature-dependent PL intensity (*I*) is the main variable, along with the density of quenching centers (*A*) and the strength of electron–phonon coupling (*C*). The thermal quenching process is characterized by the thermal activation energy (*E*), and the Boltzmann constant (*k_B_*) is utilized in the formula to relate temperature and energy. The thermal activation energies of MoS_2_ (82.6 meV) and WSe_2_ (61.5 meV) were determined via Arrhenius fitting of the experimental data presented in Figure 4c,d. The two materials exhibit different thermal activation energies, *E*, due to differences in the bandgap, effective mass, and mechanical strain. The distinct crystal structures and chemical bonding may also contribute to different effective masses in the MoS_2_/WSe_2_ heterostructure, resulting in varying carrier mobilities and activation energies. The observed differences in the thermal activation energies between MoS_2_ and WSe_2_ can ultimately be attributed to material-specific factors, including band structures, carrier dynamics, and mechanical forces. As shown in Figure 4e,f, the photoluminescence peak position, corresponding to the direct excitonic transition energy in MoS_2_/WSe_2_, is affected by temperature variations. An increasing temperature causes a redshift in the PL peak, indicating a reduction in the energy gap of the heterostructure [37]. This energy gap refers to the energy of the lowest allowable exciton state, which corresponds to the energy required to create an electron–hole pair in the system. The decreased energy gap shifts the lowest allowable exciton state to lower energy. This effect is commonly observed in various materials, including semiconductors and insulators. Density functional theory (DFT) calculations are performed in Supplementary Section S4. The relationship between temperature and the PL peak position is typically described by empirical equations, such as the one proposed by Varshni [38], given in Equation (2):

(2) $$E_{g}\left(T\right)=E_{g}^{0}+\alpha \frac{T^{2}}{T+\beta }$$

In this equation, $E_{g}^{0}$ represents the band gap at a temperature of absolute zero, where *T* = 0 K; *β* is a constant approximating the Debye temperature of the material; and *α* is the coefficient representing the change in bandgap energy with temperature. The Varshni fitting shown in Figure 4e,f provides a good agreement with the experimental data, yielding bandgap values of *E*_0_ = 1.94 eV and 1.64 eV for MoS_2_ and WSe_2_, respectively. In the same way, the O’Donnell and Chen formula, as given in Equation (3), is another model to fit the temperature-dependent PL and obtain the bandgap of the material [39].

(3) $$E_{g}=E_{g}^{0}-S<\frac{h}{2\pi }>\left[\cot\left(\frac{h\omega }{4\pi k_{B}T}\right)-1\right]$$

Here, the parameter $E_{g}^{0}\;$ gives the bandgap at zero temperature, while *S* gives information on electron–phonon coupling, and *ω* is the photon frequency. The fitting curves in Figure 4e,f for both MoS_2_ and WSe_2_ peak position shifting versus temperature are well-matched to the experimental data. The band gap values obtained using the O’Donnell and Chen formula for MoS_2_ and WSe_2_ are 1.93 eV and 1.65 eV, respectively, which are consistent with the results from the Varshni fitting, as well as with previously reported observations [40]. These temperature-dependent observations of phonon scattering and the interlayer coupling of heterostructures provide a significant influence on the performance of heterostructure devices; for example, the thermal noise is serious in photodetectors, and the carrier mobility of transistors is highly influenced by the environmental temperature.

## 4. Conclusions

Our study provides important insights into the temperature-dependent photoluminescence and bandgap characteristics of the MoS_2_/WSe_2_ heterostructure. These findings expand our understanding of this material system and have potential implications for various technological applications. We observed that both the longitudinal and transverse modes of MoS_2_ and WSe_2_ exhibit a linear variation in peak position and full width at half maximum with temperature. The quenching of the photoluminescence in the heterostructure is attributed to non-radiative recombination mechanisms. The behavior of the photoluminescence with temperature is similar to that observed in bulk MoS_2_ and WSe_2_. Furthermore, we found that differences in the bandgap, effective mass, and mechanical strain of MoS_2_ and WSe_2_ lead to different thermal activation energies, with values of 82.6 meV and 61.5 meV, respectively. Overall, our study contributes to the existing knowledge based on MoS_2_/WSe_2_ heterostructures and provides important insights into their temperature-dependent optoelectronic properties, which can enlighten the design and optimization of various devices.

## Data Availability

Data will be made available on request.

## Supplementary Materials

The following supporting information can be downloaded at: https://www.mdpi.com/article/10.3390/nano13162349/s1: Section S1. CVD preparation of MoS_2_ and WSe_2_ and their corresponding AFM analysis. Section S2. Preparation of MoS_2_/WSe_2_ heterostructure. Section S3. Experimental setup of Raman and PL spectroscopy. Section S4. PL Measurement and Details of the DFT Calculation of MoS_2_/WSe_2_ Hetrostructure. References [41,42,43,44,45,46,47,48,49,50] are cited in the Supplementary Materials.

## References

[1] Yin X., Tang C.S., Zheng Y., Gao J., Wu J., Zhang H., Chhowalla M., Chen W., Wee A.T. (2021). Recent developments in 2D transition metal dichalcogenides: Phase transition and applications of the (quasi-) metallic phases. Chem. Soc. Rev..

[2] Zhang Y., Chang T.-R., Zhou B., Cui Y.-T., Yan H., Liu Z., Schmitt F., Lee J., Moore R., Chen Y. (2014). Direct observation of the transition from indirect to direct bandgap in atomically thin epitaxial MoSe_2_. Nat. Nanotechnol..

[3] Radisavljevic B., Radenovic A., Brivio J., Giacometti V., Kis A. (2011). Single-layer MoS_2_ transistors. Nat. Nanotechnol..

[4] Sitt A., Hadar I., Banin U. (2013). Band-gap engineering, optoelectronic properties and applications of colloidal heterostructured semiconductor nanorods. Nano Today.

[5] Hadi M.A., Islam M.N., Podder J. (2022). Indirect to direct band gap transition through order to disorder transformation of Cs_2_AgBiBr_6_ via creating antisite defects for optoelectronic and photovoltaic applications. RSC Adv..

[6] Chaves A., Azadani J.G., Alsalman H., da Costa D.R., Frisenda R., Chaves A.J., Song S.H., Kim Y.D., He D., Zhou J. (2020). Bandgap engineering of two-dimensional semiconductor materials. NPJ 2D Mater. Appl..

[7] Singh E., Kim K.S., Yeom G.Y., Nalwa H.S. (2017). Atomically thin-layered molybdenum disulfide (MoS_2_) for bulk-heterojunction solar cells. ACS Appl. Mater. Interfaces.

[8] Tartakovskii A. (2020). Excitons in 2D heterostructures. Nat. Rev. Phys..

[9] Jadczak J., Kutrowska-Girzycka J., Schindler J.J., Debus J., Watanabe K., Taniguchi T., Ho C.-H., Bryja L. (2021). Investigations of electron-electron and interlayer electron-phonon coupling in van der Waals hBN/WSe_2_/hBN heterostructures by photoluminescence excitation experiments. Materials.

[10] Qiao H., Liu H., Huang Z., Hu R., Ma Q., Zhong J., Qi X. (2021). Tunable electronic and optical properties of 2D monoelemental materials beyond graphene for promising applications. Energy Environ. Mater..

[11] Kim H., Ahn G.H., Cho J., Amani M., Mastandrea J.P., Groschner C.K., Lien D.-H., Zhao Y., Ager III J.W., Scott M.C. (2019). Synthetic WSe_2_ monolayers with high photoluminescence quantum yield. Sci. Adv..

[12] Wen W., Wu L., Yu T. (2020). Excitonic lasers in atomically thin 2D semiconductors. ACS Mater. Lett..

[13] Ahmad W., Gong Y., Abbas G., Khan K., Khan M., Ali G., Shuja A., Tareen A.K., Khan Q., Li D. (2021). Evolution of low-dimensional material-based field-effect transistors. Nanoscale.

[14] Ahsan M.A., He T., Noveron J.C., Reuter K., Puente-Santiago A.R., Luque R. (2022). Low-dimensional heterostructures for advanced electrocatalysis: An experimental and computational perspective. Chem. Soc. Rev..

[15] Kanade C.K., Seok H., Kanade V.K., Aydin K., Kim H.-U., Mitta S.B., Yoo W.J., Kim T. (2021). Low-temperature and large-scale production of a transition metal sulfide vertical heterostructure and its application for photodetectors. ACS Appl. Mater. Interfaces.

[16] Do T.-N., Idrees M., Amin B., Hieu N.N., Phuc H.V., Hoa L.T., Nguyen C.V. (2020). First principles study of structural, optoelectronic and photocatalytic properties of SnS, SnSe monolayers and their van der Waals heterostructure. Chem. Phys..

[17] Kim S.E., Mujid F., Rai A., Eriksson F., Suh J., Poddar P., Ray A., Park C., Fransson E., Zhong Y. (2021). Extremely anisotropic van der Waals thermal conductors. Nature.

[18] Li F., Feng Y., Li Z., Ma C., Qu J., Wu X., Li D., Zhang X., Yang T., He Y. (2019). Rational kinetics control toward universal growth of 2D vertically stacked heterostructures. Adv. Mater..

[19] Halim N.D., Zaini M.S., Talib Z.A., Liew J.Y.C., Kamarudin M.A. (2022). Study of the electron-phonon coupling in PbS/MnTe quantum dots based on temperature-dependent photoluminescence. Micromachines.

[20] Vaquero D., Salvador-Sánchez J., Clericò V., Diez E., Quereda J. (2022). The low-temperature photocurrent spectrum of monolayer MoSe_2_: Excitonic features and gate voltage dependence. Nanomaterials.

[21] Yamada Y., Yoshimura T., Ashida A., Fujimura N., Kiriya D. (2021). Strong photoluminescence enhancement from bilayer molybdenum disulfide via the combination of UV irradiation and superacid molecular treatment. Appl. Sci..

[22] Zhao K., He D., Fu S., Bai Z., Miao Q., Huang M., Wang Y., Zhang X. (2022). Interfacial coupling and modulation of van der Waals heterostructures for nanodevices. Nanomaterials.

[23] Wu H., Lin M.-L., Leng Y.-C., Chen X., Zhou Y., Zhang J., Tan P.-H. (2022). Probing the interfacial coupling in ternary van der Waals heterostructures. NPJ 2D Mater. Appl..

[24] Kumar A., Kumar V., Romeo A., Wiemer C., Mariotto G. (2021). Raman spectroscopy and in situ XRD probing of the thermal decomposition of Sb_2_Se_3_ thin films. J. Phys. Chem. C.

[25] Ono R., Imai S., Kusama Y., Hamada T., Hamada M., Muneta I., Kakushima K., Tsutsui K., Kano E., Ikarashi N. (2022). Elucidation of PVD MoS_2_ film formation process and its structure focusing on sub-monolayer region. Jpn. J. Appl. Phys..

[26] Arafat A., Islam M.S., Ferdous N., Islam A.J., Sarkar M.M.H., Stampfl C., Park J. (2022). Atomistic reaction mechanism of CVD grown MoS_2_ through MoO_3_ and H_2_S precursors. Sci. Rep..

[27] Teli A., Beknalkar S., Mane S., Bhat T., Kamble B., Patil S., Sadale S., Shin J. (2022). Electrodeposited crumpled MoS_2_ nanoflakes for asymmetric supercapacitor. Ceram. Int..

[28] Zheng X., Zhu Y., Sun Y., Jiao Q. (2018). Hydrothermal synthesis of MoS_2_ with different morphology and its performance in thermal battery. J. Power Sources.

[29] Huang M., Ali W., Yang L., Huang J., Yao C., Xie Y., Sun R., Zhu C., Tan Y., Liu X. (2023). Multifunctional optoelectronic synapses based on arrayed MoS_2_ monolayers emulating human association memory. Adv. Sci..

[30] Jia H., Yang R., Nguyen A.E., Alvillar S.N., Empante T., Bartels L., Feng P.X.-L. (2016). Large-scale arrays of single-and few-layer MoS_2_ nanomechanical resonators. Nanoscale.

[31] Li H., Zhang Q., Yap C.C.R., Tay B.K., Edwin T.H.T., Olivier A., Baillargeat D. (2012). From bulk to monolayer MoS_2_: Evolution of Raman scattering. Adv. Funct. Mater..

[32] Zeng H., Liu G.-B., Dai J., Yan Y., Zhu B., He R., Xie L., Xu S., Chen X., Yao W. (2013). Optical signature of symmetry variations and spin-valley coupling in atomically thin tungsten dichalcogenides. Sci. Rep..

[33] Ramasubramaniam A. (2012). Large excitonic effects in monolayers of molybdenum and tungsten dichalcogenides. Phys. Rev. B.

[34] Jelken J., Lambin C.D., Avilés M.A.O., Lagugné-Labarthet F. (2023). Real-time observation of photo-oxidation of single MoS_2_ flakes using stochastic optical reconstruction microscopy. J. Phys. Chem. C.

[35] Di Bartolomeo A., Kumar A., Durante O., Sessa A., Faella E., Viscardi L., Intonti K., Giubileo F., Martucciello N., Romano P. (2023). Temperature-dependent photoconductivity in two-dimensional MoS_2_ transistors. Mater. Today Nano.

[36] Wu Z.Y., Zhuang J.-H., Lin Y.-T., Chou Y.-H., Wu P.C., Wu C.-L., Chen P., Hsu H.-C. (2021). One-and two-photon excited photoluminescence and suppression of thermal quenching of CsSnBr_3_ microsquare and micropyramid. ACS Nano.

[37] Chen M., Zhou B., Wang F., Xu L., Jiang K., Shang L., Hu Z., Chu J. (2018). Interlayer coupling and the phase transition mechanism of stacked MoS_2_/TaS_2_ heterostructures discovered using temperature dependent Raman and photoluminescence spectroscopy. RSC Adv..

[38] Varshni Y.P. (1967). Temperature dependence of the energy gap in semiconductors. Physica.

[39] O’donnell K.P., Chen X. (1991). Temperature dependence of semiconductor band gaps. Appl. Phys. Lett..

[40] Hu Z., Bao Y., Li Z., Gong Y., Feng R., Xiao Y., Wu X., Zhang Z., Zhu X., Ajayan P.M. (2017). Temperature dependent Raman and photoluminescence of vertical WS_2_/MoS_2_ monolayer heterostructures. Sci. Bull..

[41] Liu B., Fathi M., Chen L., Abbas A., Ma Y., Zhou C. (2015). Chemical vapor deposition growth of monolayer WSe_2_ with tunable device characteristics and growth mechanism study. ACS Nano.

[42] Yao Z., Liu J., Xu K., Chow E.K., Zhu W. (2018). Material synthesis and device aspects of monolayer tungsten diselenide. Sci. Rep..

[43] Kresse G., Furthmüller J. (1996). Efficiency of ab-initio total energy calculations for metals and semiconductors using a plane-wave basis set. Comput. Mater. Sci..

[44] Blöchl P.E. (1994). Projector augmented-wave method. Phys. Rev. B.

[45] Perdew J.P., Burke K., Ernzerhof M. (1996). Generalized gradient approximation made simple. Phys. Rev. Lett..

[46] Grimme S., Antony J., Ehrlich S., Krieg H. (2010). A consistent and accurate ab initio parametrization of density functional dispersion correction (DFT-D) for the 94 elements H-Pu. Chem. Phys..

[47] Li Y., Li Y.-L., Araujo C.M., Luo W., Ahuja R. (2013). Single-layer MoS_2_ as an efficient photocatalyst. Catal. Sci. Technol..

[48] Zhang L., Huang L., Yin T., Yang Y. (2021). Strain-induced tunable band offsets in blue phosphorus and WSe_2_ van der Waals heterostructure. Crystals.

[49] Su X., Ju W., Zhang R., Guo C., Zheng J., Yong Y., Li X. (2016). Bandgap engineering of MoS_2_/MX_2_ (MX_2_ = WS_2_, MoSe_2_ and WSe_2_) heterobilayers subjected to biaxial strain and normal compressive strain. RSC Adv..

[50] Chiu M.-H., Zhang C., Shiu H.-W., Chuu C.-P., Chen C.-H., Chang C.-Y.S., Chen C.-H., Chou M.-Y., Shih C.-K., Li L.-J. (2015). Determination of band alignment in the single-layer MoS_2_/WSe_2_ heterojunction. Nat. Commun..

