# Supplementary material for: Temperature-Dependent Phonon Scattering and Photoluminescence in Vertical MoS2/WSe2 Heterostructures
Source: Nanomaterials (Basel). 2023 Aug 16;13(16):2349. doi: 10.3390/nano13162349 (PMC10459064; doi:10.3390/nano13162349)
Supplement: Supplementary file 1 [file nanomaterials-13-02349-s001.zip › nanomaterials-2545894-supplementary.pdf]

nanomaterials

Supplementary Materials

Temperature-Dependent Phonon Scattering and Photoluminescence in Vertical MoS_2/WSe_2 Heterostructures

Wajid Ali [1], Ye Liu [1], Ming Huang [1], Yunfei Xie [1] and Ziwei Li [1,2,*]

[1] Hunan Institute of Optoelectronic Integration, College of Materials Science and Engineering, Hunan University, Changsha 410082, China; wajidali@hnu.edu.cn (W.A.)
[2] Wuhan National Laboratory for Optoelectronics, School of Physics, Huazhong University of Science and Technology, Wuhan 430074, China
* Correspondence: ziwei_li@hnu.edu.cn

S1. CVD preparation of MoS_2 and WSe_2 and their corresponding AFM analysis

Chemical vapor deposition is a widely used technique for the production of two-dimensional (2D) materials such as MoS_2 and WSe_2. The experimental setup for CVD preparation of MoS_2 and WSe_2 generally comprises a vacuum pump, simple furnace, quartz tube, and a precursor source of the desired material, as illustrated in schematic Figure S1a. The standard silicon wafer substrate was cleaned with acetone and deionized water, followed by drying under nitrogen gas flow. Next, MoO_3 and S precursors were placed into the boats as shown in schematic Figure S1a. The temperature was gradually increased over a period of 30 minutes to 810°C and maintained at that temperature for 10 minutes. Sulphur, carried by argon Ar gas from the right side, combined with MoO_3 vapours to form triangular MoS_2 on the inverted silicon substrate at the second boat. Subsequently, the system was allowed to naturally cool down to room temperature. Optical microscopy confirmed the successful growth of MoS_2 as shown in Figure S1b. The synthesis of WSe_2 was employed by using WO_3 and Se precursor at an elevated temperature of 950°C, which is similar as previous reports [1,2]. An optical microscopic image of a single layer WSe_2 is shown in Figure S1c. Monolayered MoS_2 and WSe_2, as well as their hetrostructure were analyzed by contact mode analysis via atomic force microscope (AFM) as shown in Figure S1d. The height of heterostructure is determined as 1.89 nm.

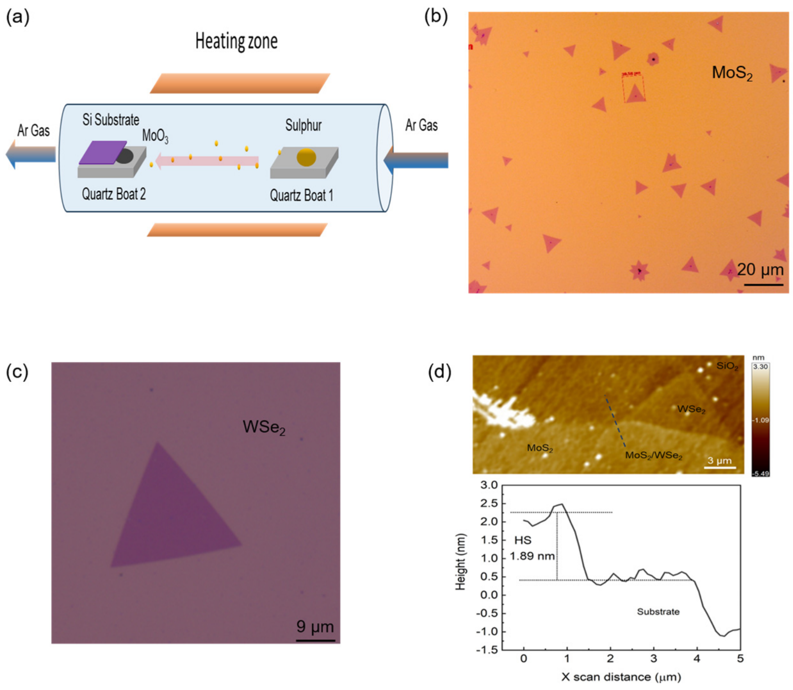

Figure S1. (a) The schematic of CVD preparation of MoS_2 monolayers and (b, c) The optical microscope images of monolayer MoS_2 and WSe_2 respectively. (d) Atomic force microscopy images of hetrostructure with corresponding height profile.

S2. Preparation of MoS_2/WSe_2 heterostructure

To transfer the CVD-grown MoS_2 monolayer to the already-grown WSe_2 monolayer on a silicon substrate, a simple water drop method [3] was employed. The MoS_2 monolayers were initially lifted off the growth substrate onto a PDMS (Polydimethylsiloxane) stamp mounted on a glass slide with the assistance of DI (deionized) water injected to wet the SiO_2/MoS_2/PDMS interface. The PDMS stamp carrying the MoS_2 monolayers was then dried with N_2 gas. Following this, a single piece of monolayer on the PDMS was carefully chosen and separated from others through knife cutting. Likewise, a single piece of WSe_2 was located on the target substrate. The PDMS stamp with the MoS_2 monolayers was then used to dry-transfer the MoS_2 onto the WSe_2 on the substrate. The PDMS was lifted in the direction marked by the arrow, and the MoS_2 remained on the WSe_2 due to van der Waals adhesion. The heterostructure was subjected to annealing at 70 °C for 12 hours in a vacuum furnace to improve its crystallinity by removing any water molecules present. The as-prepared heterostructure experimental setup, along with all the associated characterization techniques, is discussed in the primary text. The schematic representation of the transfer process and hetrostructure preparation is described in Figure S2.

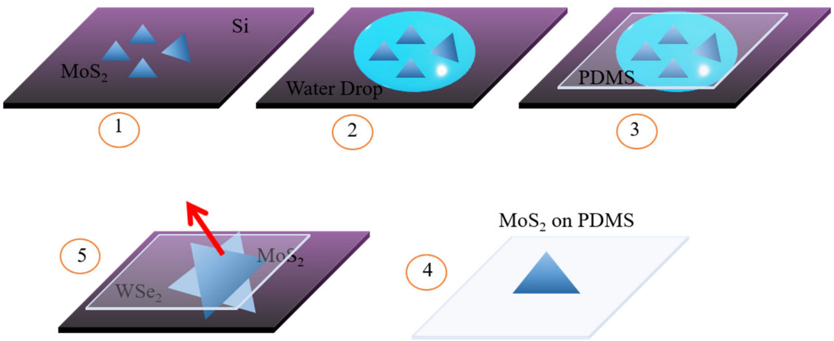

Figure S2. Schematic of heterostructure preparation by water drop transfer via PDMS.

S3. Experimental setup of Raman and PL spectroscopy

Figure S3 presents the experimental setup used to investigate the heterostructure structure. To do so, the samples were imaged with a CCD camera positioned behind a microscope objective lens with a magnification of 50×. White light was used to illuminate the samples. Subsequently, the same microscope objective lens was employed to irradiate the sample with a 532 nm-wavelength laser, which served as the excitation source for Raman and photoluminescence spectroscopy. The irradiation power of the laser was carefully controlled (to 1.2 mW) during the measurements. The Raman and PL signals emitted by the samples were collected by the microscope objective and directed towards a spectrometer, which isolated them from the excitation beam using a notch filter. The experimental results are presented in Figure 2 and Figure 3 of the main text.

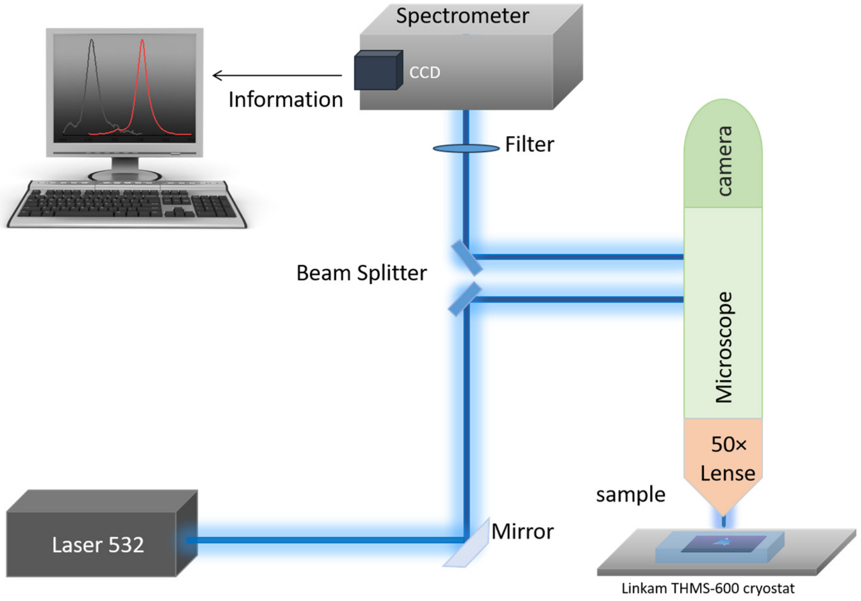

Figure S3. Schematic diagram of the microscopy–spectroscopy of the Raman and PL measurement.

S4. PL Measurement and Details DFT Calculation of MoS_2/WSe_2 Hetrostructure

Figure S4a and 4b depict measurements of the photoluminescence in both the monolayer position (region 1, region 2) and heterostructure region 3 of MoS_2 and WSe_2 at room temperature. Specifically, in the heterostructure region 3, a significant reduction in peak intensity is observed compared to the MoS_2 and WSe_2 monolayer regions. This intriguing observation can be attributed to the presence of atomically thin MoS_2/WSe_2 heterostructures, which display remarkable ultrafast charge transfer capabilities. The ultrafast charge transfer process within the MoS_2/WSe_2 heterostructures efficiently quenches the photoluminescence in the heterostructure region. This phenomenon stems from the effective capture and localization of charge carriers, preventing their recombination and subsequent light emission. Further elucidation can be obtained from the following DFT results.

Computational Method applied in DFT calculaiton

All calculations were carried out employing the Vienna ab initio package (VASP) [4], which incorporates the projector augmented-wave (PAW) method [5] to handle electron-ion interactions. The Perdew-Burke-Ernzerhof (PBE) [6] version of generalized gradient approximation (GGA) was adopted here for the exchange-correlation interaction, with the energy cutoff set at 500 eV. Simultaneously, DFT with dispersion correction (DFT-D3) [7]

described the vdW interaction of the layers. The crystal structures used in our calculations were optimized until the energy and force convergence were 10^{-5} eV and 0.01 eV/Å, respectively. To mitigate or reduce the influence of neighboring replicas, a vacuum layer of 18 Å units along the c-axis has been introduced to calculate 2D structures. For electronic property calculations, a Γ-centered 9 × 9 × 1 Monkhorst-Pack mesh is used for sampling the first Brillouin zone.

Result and Discussion

Initially, we studied the electronic properties of isolated MoS_2 and WSe_2 nanosheets before investigating the MoS_2/WSe_2 vdW heterostructure. The electronic band structure calculations of MoS_2 and WSe_2 nanosheets are shown in Figure S4c, d. The MoS_2 nanosheet is a direct bandgap (1.74 eV) semiconductor at K-point, which is more in line with our experimental and earlier results [8]. Similarly, the WSe_2 nanosheet also exhibits a direct bandgap (1.64 eV) at K-point. Its bandgap value is consistent with the present experimental and existing literature [9]. While the designed MoS_2/WSe_2 vdW heterostructure demonstrates the direct band gap (0.66 eV) where both conduction band minimum (CBM) and valence band maximum (VBM) are at K-points (Figure S4e). The calculated band gap result is consistent with previous findings [8-10]. The valence band maximum composed of MoS_2 whereas the conduction band minimum dominates by WSe_2 which indicates the MoS_2/WSe_2 vdW heterojunction has a typical type-II band alignment [11]. The inherent type-II band alignment facilitates the separation of photogenerated carriers. The MoS_2/WSe_2 vdW heterojunction modified the optical and electronic properties, potentially leading to enhanced excitonic effects, PL and the ability to tailor optoelectronic applications.

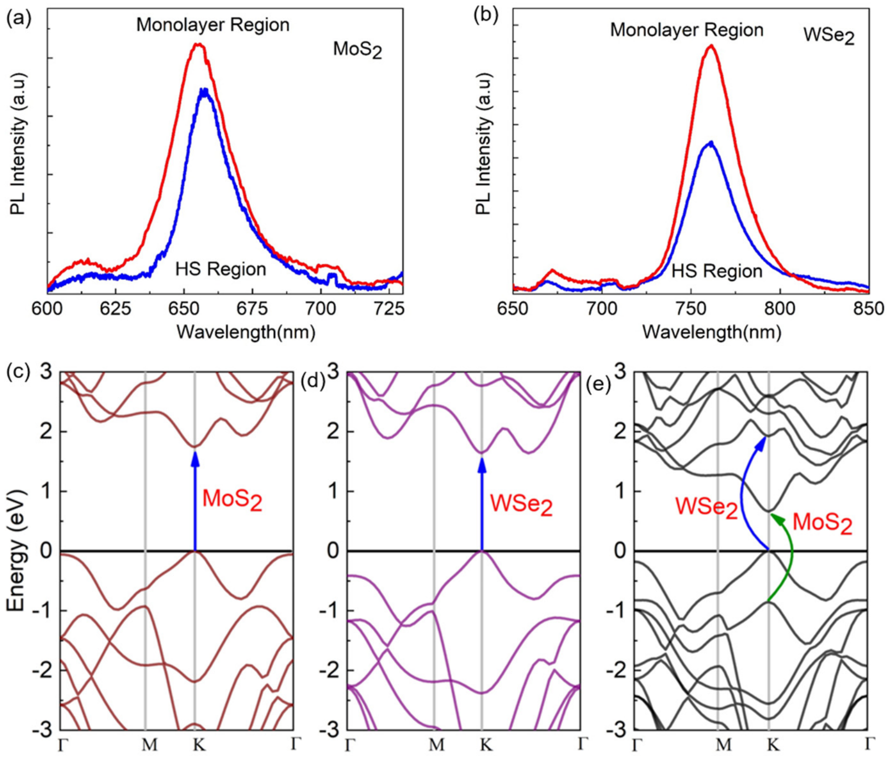

Figure S4. (a, b) PL measurement at monolayer and HS positions for both MoS_2 and WSe_2 respectively. (c) Single-layer MoS_2 and (d) WSe_2 as well as (e) their heterostructure. The arrows indicate the lowest energy transitions between two layered materials. For all three observation the Fermi levels are standardized at 0 eV.

References

1. Liu, B.; Fathi, M.; Chen, L.; Abbas, A.; Ma, Y.; Zhou, C. Chemical vapor deposition growth of monolayer WSe_2 with tunable device characteristics and growth mechanism study. *ACS Nano* **2015**, *9*, 6119–6127.
2. Yao, Z.; Liu, J.; Xu, K.; Chow, E.K.; Zhu, W. Material synthesis and device aspects of monolayer tungsten diselenide. *Sci. Rep.* **2018**, *8*, 5221.
3. Jia, H.; Yang, R.; Nguyen, A.E.; Alvillar, S.N.; Empante, T.; Bartels, L.; Feng, P.X.-L., Large-scale arrays of single-and few-layer MoS_2 nanomechanical resonators. *Nanoscale* **2016**, *8*, 10677–10685.
4. Kresse, G.; Furthmüller, J., Efficiency of ab-initio total energy calculations for metals and semiconductors using a plane-wave basis set. *Comput. Mater. Sci.* **1996**, *6*, 15–50.
5. Blöchl, P.E., Projector augmented-wave method. *Phys. Rev. B.* **1994**, *50*, 17953.
6. Perdew, J.P.; Burke, K.; Ernzerhof, M. Generalized gradient approximation made simple. *Phys. Rev. Lett.* **1996**, *77*, 3865.
7. Grimme, S.; Antony, J.; Ehrlich, S.; Krieg, H. A consistent and accurate ab initio parametrization of density functional dispersion correction (DFT-D) for the 94 elements H-Pu. *Chem. Phys.* **2010**, *15*, 132.
8. Li, Y.; Li, Y.-L.; Araujo, C.M.; Luo, W.; Ahuja, R. Single-layer MoS_2 as an efficient photocatalyst. *Catal. Sci. Technol.* **2013**, *3*, 2214–2220.
9. Zhang, L.; Huang, L.; Yin, T.; Yang, Y. Strain-induced tunable band offsets in blue phosphorus and WSe_2 van der Waals heterostructure. *Crystals.* **2021**, *11*, 470.
10. Su, X.; Ju, W.; Zhang, R.; Guo, C.; Zheng, J.; Yong, Y.; Li, X. Bandgap engineering of MoS_2/MX_2 (MX_2= WS_2, $MoSe_2$ and WSe_2) heterobilayers subjected to biaxial strain and normal compressive strain. *RSC Adv.* **2016**, *6*, 18319–18325.
11. Chiu, M.-H.; Zhang, C.; Shiu, H.-W.; Chuu, C.-P.; Chen, C.-H.; Chang, C.-Y. S.; Chen, C.-H.; Chou, M.-Y.; Shih, C.-K.; Li, L.-J. Determination of band alignment in the single-layer MoS_2/WSe_2 heterojunction. *Nat. Commun.* **2015**, *6*, 7666.

.
